# A national mental health cascade training programme for practitioners supporting unaccompanied minors in Greece

**DOI:** 10.1177/13591045241242324

**Published:** 2024-03-23

**Authors:** Panos Vostanis, Panagiotis Sofios, Alexandra Petrali, Michelle O’Reilly

**Affiliations:** 1 Department of Media, Communication and Sociology, 4488 University of Leicester, UK; 2 560351 SOS Children’s Villages Greece, Athens, Greece

**Keywords:** Child, young people, refugee, asylum seeking, unaccompanied, mental health, training, train-the-trainer, capacity building

## Abstract

**Background:**

Practitioners who support unaccompanied minors (UAMs) come from different professional backgrounds and often are not appropriately trained to address children’s complex mental health needs. This gap informed a training programme across all accommodation centres in Greece.

**Methods:**

The aim of the Train-of-Trainer (ToT) national programme was to upskill trainers from 17 organisations to cascade knowledge. Training was interprofessional, trauma-informed and culturally sensitive. A pilot implementation involved 199 practitioners from all disciplines. A sub-sample of 33 practitioners, nine managers and six trainers shared their experiences in focus group discussions, which were analysed through a thematic framework.

**Results:**

Participants found that the programme was useful in generating knowledge relevant to children’s needs and their roles, addressing the emotional impact of trauma on staff, sharing learning across professional disciplines and being interactive, but systemic support should be put in place for benefits to be sustained.

**Conclusions:**

Train-of-Trainer is a resource-effective approach to upskill mixed staff groups across many organisations. This should involve caregivers and staff with limited educational opportunities, while accommodating for different professional needs. Training should be integrated to service budgets, specifications and structures.

## Plain language summary

Staff who support refugee children without parents come from different backgrounds, and often have limited mental health knowledge and skills. To address this gap, a national programme was established for 17 organisations hosting accommodation centres across Greece. Selected professionals were upskilled as trainers to cascade knowledge to their staff group. Training was based on the understanding of the impact of trauma and children’s cultural needs and was provided to interdisciplinary staff groups. In total, 199 staff attended pilot training. Experiences of the training were shared by a sub-group of 33 trained staff, nine managers and six trainers through focus group discussions. These were analysed through a qualitative thematic approach. Key findings included the relevance of new knowledge to children’s needs and staff roles, training as a team, using learning methods based on real issues, and supports that should be put in place to ensure that benefits are maintained. Such a model of cascading knowledge and skills can be useful for large numbers of organisations and staff supporting refugee and other vulnerable children. Training should include staff such as caregivers, who may have had limited educational opportunities, while balancing between different staff roles. Training should be integral to all functions of accommodation centres.

## Introduction

Refugee children often have complex mental health needs, which are associated with multiple risk factors during the pre-, peri- and post-migration stages (Scharpf et al., 2020). Vulnerabilities are compounded for unaccompanied minors (UAMs) because of loss of primary caregivers, maltreatment, labour or sexual exploitation (Jensen et al., 2019). Despite knowledge regarding risk factors, UAMs face several barriers in accessing mental health care. Established reasons are related to their transitional life circumstances, conceptualisation of mental health, stigma, mistrust, legal uncertainty, and prioritisation of basic needs; as well as systemic challenges such as lack of culturally sensitive interventions, care pathways, and adequately skilled providers (Eruyar et al., 2022). UAMs often reported lack of information and understanding of mental health care, misidentification with the legal process, and lack of engagement because of stigma and cultural factors (Demazure et al., 2022).

To this effect, mental health professionals may lack contextual knowledge, while other health and social care professionals are not usually sufficiently trained to enhance psychosocial support within their roles (Due & Currie, 2022). As UAMs are supported by different community and residential agencies, integration of mental health to existing care is essential, because mental health and social care needs are inter-linked. It also provides an opportunity, because these agencies have substantial – albeit fragmented – contact with this population across various settings. For these reasons, several training programmes and approaches have been reported for interprofessional groups. These variably found improvement in knowledge, confidence, skills such as empathy or listening, and recognition of mental health problems, but were inconclusive on impact on staff practice (Guajardo et al., 2018; McDonald et al., 2021).

Training in relation to refugee mental health has been informed by several frameworks such as cross-cultural and trauma-informed theories (Im & Swan, 2020; Ward, 2022). Time-limited approaches for volunteers or professionals with minimal baseline psychosocial skills were, however, shown to have little impact on capacity-building (Horn et al., 2019). Consequently, training has been influenced by the Train-of-Trainer (ToT) or cascade approach, which is based on upskilling a group of professionals, who subsequently transfer knowledge and skills to their interprofessional team or local workforce. Overall, ToT approaches of interactive and multifaceted delivery, which were blended with topic-related modules, were shown to be more effective in transferring knowledge to health and social care professionals than individual learning (Pearce et al., 2012). An additional lasting benefit can be the redistribution of mental health interventions between mental health and other professionals (Fragkiadaki et al., 2020).

Capacity-building is particularly important for countries with a continuous influx of asylum-seeking children because of their geopolitical position. Greece is located on the southern east border of Europe, with many sea crossings from Asia and northern Africa, and is often faced with a response crisis and demand for protection and services for children seeking asylum or, more often, in transition to northern Europe (UNICEF, 2020). Support systems are insufficient to respond to this need because of limited infrastructure and skilled professionals, funding, and access to mental health interventions. This service gap led to the development of a national child mental health capacity-building programme.

## Methods

The research aim was to establish stakeholders’ (staff, trainers and managers) perspectives of the implementation of a national child mental health cascade training programme in relation to unaccompanied minors in Greece. This aim was addressed through two research questions:

(a) What was the perceived impact on service providers of a national ToT child mental health programme for unaccompanied minors?
(b) Which were the enabling and hindering factors in the implementation process?

### Context

Numbers of UAMs continuously fluctuate, depending on policies and external factors, especially war conflicts. Such fluctuation influences accommodation places and supporting budgets. In Greece, all issues related to UAMs are co-ordinated by the Special Secretariat for the Protection of UAMs. These include guardianship, needs assessment, child protection, placement, legal process, service co-ordination, and standards for accommodation centres. Between 2016–2023 44,920 UAMs arrived in Greece, mainly from Afghanistan (25%), Pakistan (14%) and Syria (9%). During the year of this study (2022), there were 2,624 officially classified UAMs across Greece, of whom 1,736 lived in medium-to long-term accommodation centres in the mainland, and which were the focus of the programme (SSP for UAMs, 2023). The remaining UAMs lived in reception, emergency and semi-independent living settings in islands and the mainland. The majority were between 14-18 years (90%) and male (83%).

Accommodation centres are provided by non-governmental organisations (NGOs). In response to government guidelines, each centre is typically staffed by an interdisciplinary team, including caregivers, administrators, cooks, domestics, educationalists, social workers and psychologists, as well as access to interpreters and legal advisers. Some centres had arranged staff supervision and child psychiatry input, whilst the majority referred to the local child and adolescent mental health service (CAMHS). Key reported staff challenges were the high turnover of UAMs, timely planning, and integrated care involving external agencies (Ntagka & Cochliou, 2023). Of the 22 NGOs that hosted 72 accommodation centres with a total 1,083 staff, 20 NGOs and their 69 centres agreed to participate in the cascade or ToT programme.

Care providers were consulted through four focus groups on the content and format of the ToT, and on selection criteria for the trainers. Seventeen consultees were involved, out of 40 invited. These consisted of caregivers (3), social workers (8), psychologists (2), educationalists (2), sociologist (1) and administrator (1). Each of the 20 NGOs subsequently selected one professional to act as trainer. Of the 20 identified trainers, three did not complete the programme because of various work pressures (new job, other commitments). The 17 remaining trainers had professional background in psychology (8), social work (7) and education (2). They operated in the Athens (10), central (2), northern (2), northwest (2) and island (1) regions.

### Train-of-trainer (ToT) child mental health programme

Consistent with the literature, the programme was informed by cross-cultural trauma-informed (Im & Swan, 2020) and interprofessional education (Merriman et al., 2020) theories, in conjunction with the child refugee mental health literature (Scharpf et al., 2020). Following co-production with stakeholders, the ToT had previously been piloted, implemented and evaluated in several majority world (also referred to as low- and middle-income) countries in relation to different groups of vulnerable children, including refugee and UAMs (Eruyar et al., 2023; Vostanis et al., 2019).

**Figure 1.**
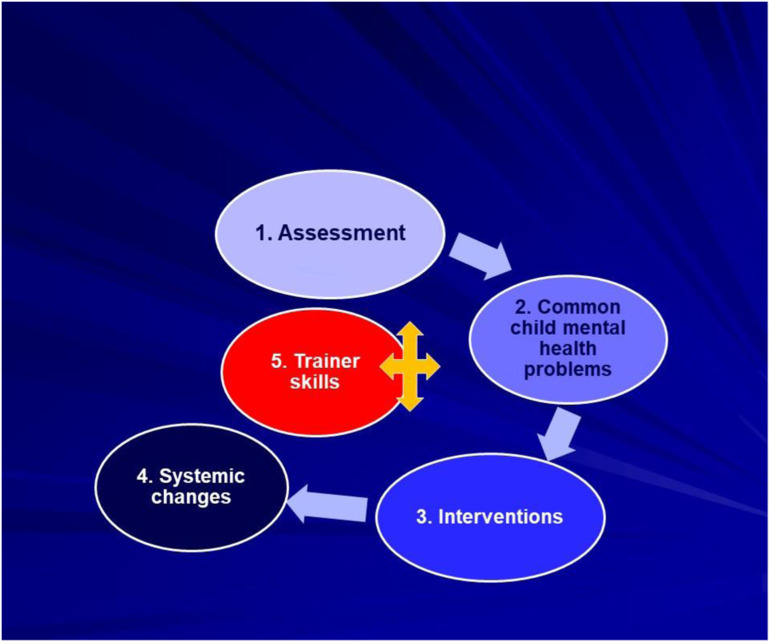
National Train-the-Trainer child mental health programme for practitioners supporting unaccompanied minors in Greece.

For the purpose of this programme, the ToT was tailored to four bimonthly modules, each consisting of weekly three-hour sessions. The eight sessions of each module included child mental health topics (four), trainer skills (two), practice of pilot training (one - see details below), and reflection (one). The themes of the four modules were, assessment (cultural concepts and stigma, impact of trauma on child development, risk and protective factors); common child mental health problems (emotional, behavioural and attachment-related); interventions (different frameworks, goal-setting, application with UAMs); and systemic changes (needs analysis, action plans, youth participation, Theory-of-Change) (Figure 1). Training involved blended approaches of theory- and evidence-informed learning, case-based and experiential activities. Supporting materials included a trainer manual, two e-learn modules, psychoeducational resources, and selected bibliography. The ToT was facilitated by the first author (PV) of child psychiatry background through remote sessions, with bimonthly visits to participating centres. The remaining roles of the research team were co-ordination of the programme without direct involvement in the training (PS), facilitation of focus groups (AP) and independent evaluation (MoR). The ToT was overseen by a Ministry-led advisory group, with additional quarterly meetings with NGO managers.

### Trainers’ pilot training with interprofessional staff groups

Trainers piloted the modules within their accommodation centres. The framework was common across all NGOs, however, trainers tailored delivery approaches to their staff group, for example following internal consultation. All training was delivered to interprofessional staff groups, as this was a key objective of the programme, but the number of staff and sessions varied, as this often reflected the variable size of participating NGOs and their accommodation centres. This was broadly representative of the workforce, rather than ‘just’ psychologists, social workers and educationalists (Table 1). Although all 17 trainers completed the ToT, three trainers did not pilot the training, for various organisational reasons such as staff compliance or other overlapping educational activities. Pilot training activities were, therefore, completed and recorded by 14 trainers.

**Table 1.** Pilot training activities across 14 participating NGOs and their accommodation centres.

Sessions	Total = 50 Mean = 4 Median = 4 Range = 1–10
Staff trained	Total = 199^a^ Mean = 15 Median = 10 Range = 3–84^b^
Professional role represented in NGO training^c^	Psychologist: 13 Caregiver: 12 Social worker: 11 Educationalist: 10 Interpreter: 5 Legal representative: 4 Cook: 4 Domestic: 3 Administrator: 3 Nurse: 2 Driver: 1

^a^Notes Not all staff attended each session within their NGO.

^b^Outliers explained by single- or multiple-centre NGOs.

^c^All NGO provided professional role breakdown, but not all provided for specific professional roles numbers.

#### Data collection

Stakeholders in the programme shared their experiences and perspectives through focus group discussions. Focus groups can engage participants in ‘collective conversations’ in relation to their insights, and in negotiating ideas and solutions (Adler et al., 2019). The topic guide explored how mental health provision was conceptualised in relation to different roles, knowledge and skills (existing, acquired and desired) to fulfil these roles, ToT implementation process, and organisational issues. Focus groups were facilitated remotely by an independent researcher. Ethics approval was obtained from the Sociology Research Ethics Committee of Leicester University in the UK. Participants provided verbal consent at the beginning of the recorded remote sessions, as they came from different regions.

Through purposive sampling, to capture voices across the stakeholder spectrum (roles and geography), a sub-sample attended four focus groups with staff who had attended pilot training (*n* = 33), one focus group with trainers (*n* = 6), and one focus group with centre managers or co-ordinators (*n* = 9). Each focus group involved between 6-9 participants. Sessions were conducted in Greek language and were video-recorded. Participants’ professional roles are summarised in Table 2.

**Table 2.** Professional roles of focus group participants.

Practitioners who attended pilot training	Psychologist: 8 Caregiver: 7 Social worker: 6 Administrator: 4 Educationalist: 3 Interpreter: 3 Cook: 2 Total staff: 33
Managers or co-ordinators	9
Trainers	6

### Data analysis

We utilised thematic analysis (Braun & Clarke, 2006), and particularly engaged with a codebook form, to allow for conflation of inductive and deductive coding processes (Braun & Clarke, 2022). Stakeholders’ data were integrated, to explore all perspectives in relation to the research questions. Interviews were transcribed in Greek by AP, translated to English and coded by PV, and returned to MoR for validation of meaning. The codebook was revisited by the whole research team, to refine codes and resolve any discrepancies.

## Results

The established themes and subthemes are presented in Table 3. Overall, participants considered the relevance of the programme to different roles and approaches in addressing children’s needs, application of learning, impact on team working, and systemic changes required to sustain benefits. Themes are described below, with supporting excerpts. As staff attended four focus groups (FG), the FG number and profession is provided under each participant quote.

**Table 3.** Established themes and subthemes.

Themes	Subthemes
Contextual knowledge	Relevance to professional roles Application Practice framework
Learning process	Interprofessional learning Delivery approaches Emotional impact
Sustainability	Educational model Team working Systemic challenges Trainer role

### Theme 1: Contextual knowledge

Baseline knowledge and relevance of topics provided by the ToT appeared to relate differently to broadly three staff groups, psychologists, social workers/educationalists and all other staff. This challenge was apparent within groups, for example topics were usually familiar to psychologists, but the process and approach provided a new perspective to some. Trainers had to adapt accordingly, and considered how complementary training levels could be possible in future.

“…as a psychologist, I already apply what we heard in the meetings. Therefore, these [topics] were more-or-less familiar.”

FG2, Psychologist

“I remembered a few issues, so that I can apply them with new additions, new ideas.”

FG4, Psychologist

“Maybe there should be some changes…mainly for psychologists and essentially for the whole psychosocial service.”

Trainer 1

In contrast, there was a previous gap in mental health knowledge and skills for residential caregivers and other ‘non-psychosocial’ staff. For this reason, these staff groups welcomed their involvement and found the training appropriate in strengthening their practical experience. For many, this was their first educational exposure, at least to mental health topics.

“Even though caregivers are a part, a link, of the shelter between the children and the social workers or psychologists, they are a neglected part.”

FG1, Caregiver

“We are cooks, we have contact with the children. Of course, we can come across a situation in the dining area when we serve children, but there is always someone from the professional personnel to join in its management. Therefore, for us these were interesting and useful, but our role is different. The trainings definitely helped us, because we learnt some things we had not thought of before, some that we knew to an extent, we had some knowledge.”

FG3, Cook

“It helped me in the way I was thinking about certain situations, to see them through a different angle that I had not viewed before.”

FG4, Administrator

New knowledge and understanding enabled staff to deal with incidents in the centre in a different or more assured way. This was particularly important for emergencies and high risk incidents when they had to provide a quick response.

“Each time there were examples that I applied in my job. Continuously. You hear what is discussed and, when faced with the problem and crisis, you think that I need to approach the child differently, s/he has a different issue, at that moment you remember what had been discussed. I have seen it in a situation that troubled me but was resolved within five minutes.”

FG1, Caregiver

“We automatically had solutions on questions and issues about children’s behaviour, based on their experiences and the way we reacted…”

FG2, Interpreter

Participants reported that they were enabled to make more links between parallel children’s and staff relationships and dynamics. Such reflection was especially important in setting boundaries and managing aggressive outbursts, while maintaining a safe and nurturing environment.

“…there will be a need for some boundary-setting, some guidelines. Then the child feels that all their previous privileges are taken away…it also helped me to see that the relationships they possibly had in their family environment, are maybe mirrored in their relationship with the caregiver.”

FG3, Caregiver

“When there are confrontation incidents, I have noticed that, when they have a good relationship with the staff, it is somehow easier to control their aggression.”

FG3, Psychologist

Although most topics related to trauma may not have been new to staff, linking theory with practice, and using tools for assessment and interventions, were viewed as important. Caregivers in particular felt empowered with this layer of knowledge on top of their empirical experience.

“We confirmed a few practices we were using without knowing them.”

FG1, Caregiver

“It really helped me understand many things, which I used without understanding why I used them. That is, it helped me code and improve my ways of approaching them.”

FG3 Caregiver

“We put into words practices that we already had and used.”

FG2, Social worker

Both managers and trainers were conscious of this gap in evidence-based and informed decision-making. The ToT thus provided an opportunity for adopting a framework and toolsets.

“...new colleagues, as well as old ones with experience, managed through these trainings to systematise their experience, and to see that what they were doing anyway was useful.”

Manager 2

Although participants generally welcomed these new skills and applications, they positioned them within their continuing professional development, which overlaps with Theme 3 on sustainability.

“It is never-ending for me, because through this we self-improve both as professionals and as people.”

FG3, Caregiver

“…sometimes half-knowledge can even prove dangerous. Therefore, it should be made crystal clear to all staff, irrespective of role, that such a brief training period cannot cover the whole range.”

FG3, Psychologist

In summary, training was challenging in meeting diverse staff needs. This was, however, positively received, especially by professionals without previous mental health training, and who often felt excluded from education, thus undermined, despite spending all their working time with vulnerable children. The main reported benefit was being provided with a framework and tools to maximise their experience and observations.

### Theme 2: Learning process

Although all centres had staff group forums, interprofessional training opportunities were relatively new for many, mostly those without ‘psychosocial’ qualifications. Learning as a team was viewed positively by most participants, including those who were familiar with the topics, because this training enabled them to understand better different perspectives.

“I am a psychologist. Next to me sits my colleague who is interpreter of Arabic. We attended together some training groups.”

FG2, Psychologist

“Views from different disciplines were heard because each discipline has different contact with the children. And this material enabled us to reflect, to register our views…this was followed by discussion, which I consider the most positive.”

FG4, Administrator

A key reported benefit of joint training was the holistic understanding of children’s needs and behaviours, consequently integrated care planning and response. Several participants appreciated what other team members could contribute through experience, theory or techniques, even if some of these views could take them out of their comfort zone.

“Being on a shift with psychologists, social workers or educationalists, while managing crises at the same time in the centre, was completely new for them. We are talking about serious incidents. The training is not only for caregivers but also for the professional team.”

FG1, Caregiver

“They are here 24 hours and work when there may be no psychologists, educationalists. The caregivers are here all day and night. Therefore, it is important that they are familiar with certain issues.”

FG3, Educationalist

Such training culture reinforced team and joint working, which managers wanted to promote across settings. Nevertheless, they, as well as trainers, acknowledged that delivery should account for different training requirements.

“We did not separate the psychosocial team on their own, the caregivers on their own and the domestics on their own. We mixed them, so that the standards rise…some topics need adjusting for certain disciplines, while they roll better with others.”

Manager 1

Participants were exposed to various delivery approaches. They related better to interactive, experiential and case-based activities, as these enabled them to apply new knowledge immediately during the training event and subsequently in their centre. Consistent with their previously described appreciation for an underpinning framework, they valued links between theory and practice, elaborating views that only theoretical teaching was experienced as non-engaging.

“We split into groups to analyse practices of dealing with cases. In general, there was a lot of interaction beyond the theory, which I believe helps to better register issues.”

FG4, Educationalist

“For me, both the challenge and pleasure at the same time lied in experiential [activities], where everyone had their own views, and how we should put together all our ideas and shape them, so that something comprehensive comes out at the end.”

FG1, Administrator

This improved capacity to relate to everyday practice, drove managers and trainers to link training events with existing service meetings, case incidents and ‘real’ concerns in the centre, as staff could thus make more sense of new knowledge.

“This somehow is part of case management, some scenarios which had to do with mental health, and we asked different roles to contribute somehow on how this situation be managed.”

Manager 4

“Also, I think that it helps a lot to relate to an incident. For example, we had discussed an argument that happened the other day. In this way you can discuss about violence and aggression, which were part of the training themes. This is a nice opportunity, daily incidents provide an opportunity for a large discussion.”

Trainer 4

Despite receiving supervision, this training offered much needed experiential, reflective and case-based approaches to their own emotional needs and required support. Working in a constantly changing and emotionally ‘charged’ environment, as all children had suffered some degree of trauma, placed additional strain on staff.

“Burnout because of rejection…there are advantages [working in the refugee field] but is also exhausting. Therefore, it is very nice to externalise a few issues and to come back lighter.”

FG2, Psychologist

“Perhaps one of the nicest topics was in relation to mental resilience, burnout in the working environment and staff relationships, because as an administrator I do not come in contact with the residents, therefore I get am indirectly involved with issues at the centre.”

FG2, Administrator

Exchanging ideas and reflecting with colleagues from different professional backgrounds and working in different settings enabled a new kind of shared learning for many participants.

“I believe that there are several topics which are worth opening, so that we can discuss them without fear of saying something wrong…so that we have the mental reserves needed for this job.”

FG3, Caregiver

In this theme, participants predominantly considered how they valued training in an interprofessional context. This enabled them to understand each other’s role, complement skills and respond holistically to children’s needs. In terms of delivery approach, theoretical principles were appreciated, as long as these were demonstrated and practiced through case-based and experiential activities.

### Theme 3: Sustainability

Participants considered extensively future training needs following the ToT. Although there was consensus that training should be ongoing, more specific suggestions were made on an educational model that could also account for organisational challenges, in particular staff turnover. A hybrid combination of induction and continuing training was thus put forward.

“But we also have new people, new arrivals of personnel. Therefore, they need to be integrated in the already formed [training] teams.”

Trainer 3

“It would be really interesting if the training took place twice, once at the beginning of a professional’s work experience in a centre, and later after a certain time.”

FG2, Social worker

“…maybe a training cycle each year, so that old colleagues come together with new ones in the same space.”

FG4, Psychologist

Managers and trainers had initiated or were considering the integration of mental health to existing mandatory training such as on child protection. They reported that the ToT had provided them with new ideas, delivery approaches and materials.

“We have a format of monthly trainings attended by all staff, which concern child protection issues. In trainings on psychosocial issues, we have incorporated characteristics of the themes we discussed [during the ToT].

Manager 4

“We have discussed as an organisation to include [mental health] in our training section.”

Trainer 2

The interdisciplinarity and integration of training to staff forums was viewed as having direct benefits on team functioning and quality of care. Participants highlighted improved communication, clarity on professional roles and holistic case management.

“It was possible to work on communication, which often has gaps among colleagues. There was an opportunity to bridge these gaps.”

FG1, Psychologist

“There were discussions between us, that is with colleagues, with caregivers. We all got on the same page, to have the same response…”

FG3, Cook

By incorporating or adjoining training to existing staff forums in some centres, this was not experienced as distinct to everyday duties.

“It works very well for us to add a training topic to staff meetings.”

Trainer 3

Several logistical barriers were considered in ensuring staff attendance and engagement. Caregivers’ shifts, work pressures (crises, external agencies, competing meetings), and multiple centres for certain NGOs were particularly challenging for practitioners, trainers and managers.

“The only challenge is that I am on night shifts.”

FG1, Caregiver

“The time is a bit limited and pressurised…it is a bit heavy.”

Trainer 2

Practitioners expressed a clear preference for face-to-face rather than remote training, as this was experienced as more engaging and interactive. Training in neutral venues and mixing with other staff groups were other perceived advantages, although not often logistically easy, with some NGOs hosting centres in different regions.

“Training outside the working environment, in [name of city], was like a breath of fresh air that week. I would also like to host individuals from other centres in our space.”

FG2, Psychologist

“Face-to-face would be ideal, I know it is practically difficult to organise, as we come from several and different areas.”

FG4, Psychologist

“…we went to the islands to organise some extra trainings.”

Manager 1

A particular feature of the ToT was that trainers worked in the same centre as their trained colleagues, some of whom may have been more experienced. For NGOs that hosted several centres, trainers were usually, albeit not always, known to other staff groups. This new dynamic was largely viewed as positive because trainers were familiar with staff needs, but also required some adjustments in working relationships.

“It was a different experience, because you see them [trainer] daily in one way, then you have to work with them in another, to present [the training]. It was a new arrangement.”

FG2, Administrator

“…she knew how it [staff group] functions, how it is, how it behaves. Therefore, she developed a familiar environment, which was very helpful.”

FG2, Interpreter

“It is a challenge, it is not real. I am not addressing it now, I am addressing it internally, I am philosophical about it.”

Trainer 1

When participants explored reasons for feeling more comfortable with a familiar trainer, they reflected on wishing to feel safe without being criticised.

“Because we knew each other, we could even argue, express our thoughts. We would have definitely been more cautious with a stranger.”

FG3, Educationalist

“I felt more comfortable, more trusting, because I felt that what I talked about would not be judged.”

FG4, Psychologist

Here, participants considered how the training programme could be sustained in future. The main challenges concerned work pressures, staff turnover and working across different settings. These could be overcome by incorporating mental health to existing mandatory and continuing education, and by regularly providing intensive introductory courses for new staff.

## Discussion

The aim of this study was to capture stakeholders’ experiences across all accommodation providers for unaccompanied minors in Greece, following a national capacity-building programme based on the Train-of-Trainer (ToT) approach. Key themes were reported by participants across professional disciplines. These related to contextual knowledge in relation to children’s needs and staff roles, methods of sharing and transferring knowledge, interdisciplinary learning, and systemic support required to sustain benefits.

The interpretation of the findings should consider certain limitations of the study. Although the programme invited all appropriate organisations, it is likely that more engaged NGOs, trainers and staff took part. Shelters in other systems may not have access to similar professional disciplines. As discussed below, the potential impact of ToT on practice was not measured beyond professional stakeholder perspectives, and UAMs were not involved in the study.

The interprofessional staff constitution of all centres was an important training baseline in not having to justify the need for mental health training at the outset. The range of disciplines and roles brought challenges in addressing different levels of experience and skills. A key objective was to involve all staff, and this was accomplished in pilot activities. Caregivers often have extensive experience without conceptual underpinning, because of exclusion from ‘professional’ activities or disciplinary language. Even more so, this applies to staff such as interpreters, administrators, cooks, domestics and drivers, who spend a lot of time with UAMs and share incidents or disclosures, without being upskilled, thus confident, for this functionality of their role. All participants in this programme welcomed such an opportunity. Their needs are obviously different to psychosocial professionals, especially psychologists, hence different training levels are required.

Training in an interprofessional context was viewed as strengthening team working, complementing roles and leading to integrated care. The findings are consistent with key competencies in relation to refugee children, identified in a review by Due and Currie (2022), so that professionals can address complex and cultural needs, built rapport, apply holistic interventions, and collaborate with other disciplines and agencies.

Experiential, reflective and case-based activities were favoured by professionals. Barwick et al. (2012) established that focus on interactive learning rather than content was more likely to lead to practice change, although learners can initially be impatient in being shown concrete techniques (O’Neal et al., 2018). The findings support the importance of providing less qualified staff with a framework that enables them to understand theirs and others’ input, for example, the aims as well as limitations of a particular intervention. Otherwise, training may reproduce existing competencies deficits without challenging practices, translating experiential knowledge, and ultimately improving care standards.

Working in this field brings additional emotional strain on staff through children’s experiences and compassion fatigue, frequent crises, parallels between unaccompanied minors and staff turnover, demanding workloads, and risk. The experiential nature of this training indicates that it cannot be designed in silo from supervision and other types of professional support, rather that these components should be complementary (Echeverri et al., 2018). Training should be extended beyond individual practice to understanding and addressing systemic issues (module 4 in this programme) (Im & Swan, 2020), but also factoring in systemic challenges and mitigating against staff burnout. For staff to engage in training, rather than view it as additional burden, they should be involved in its co-production and monitoring.

The Train-of-Trainer cascade can be a resource-effective and sustainable approach. This study captured the first stage of this process in establishing perceptions from three stakeholder group (trainers, staff and managers). The findings indicate enhanced reach because of the broad range of staff disciplines involved (Table 1), engagement, and integration and transfer of mental health knowledge to existing practice.

## Conclusion

Globally, refugee children, especially unaccompanied minors, are temporarily cared for in settings of variable quality, staffed by caregivers and professionals with no or limited mental health training, and without access to mental health care. Capacity-building for this substantive, albeit heterogenous, workforce, should thus be a priority for international and national policy. Training should have a strategic direction, be contextualised to children’s and staff needs, and follow an interprofessional philosophy. Prolonged implementation and evaluation should establish the fidelity and validity of cascading knowledge and skills, behavioural and organisational change, and interactions between staff and children. Such evidence can inform the development of a generalisable capacity-building model and supporting tools that can be used in different contexts for refugee and other vulnerable children. A Train-of-Trainer approach can maximise resources, enhance reach, and sustain impact. Sustainability will require designated policy and budgets, protection of training time, and ongoing support for a pool of existing and new trainers.
